# Effect of Multi-Walled Carbon Nanotubes on Strength and Electrical Properties of Cement Mortar

**DOI:** 10.3390/ma14010079

**Published:** 2020-12-26

**Authors:** Elena Cerro-Prada, Rosalía Pacheco-Torres, Fernando Varela

**Affiliations:** Construction, Infrastructure and Transportation Department, Universidad Politécnica de Madrid (UPM), Alfonso XII, 3, 28014 Madrid, Spain; rosalia.pacheco@upm.es (R.P.-T.); fernando.varela@upm.es (F.V.)

**Keywords:** multi-walled carbon nanotubes, cement-based materials, flexural strength, compressive strength, electrical resistivity, activation energy

## Abstract

This work aims to investigate the effects of multi-walled carbon nanotubes (MWCNTs) on the strength and electrical properties of cement mortar. MWCNTs were added to cement mortar in four different concentrations: 0.00 wt.%, 0.01 wt.%, 0.015 wt.%, and 0.02 wt.% by the mass of cement. The consistency, density, setting time and compressive and flexural strength of mixes were tested and analyzed at 28 and 90 days curing time. Mechanical performance tests confirm an increase of 25% and 20% in the ultimate compressive and flexural strength respectively, which results from MWCNT 0.02 wt.% loading at 90 days curing time. The resistivity measurements in mortars with 0.01 and 0.015 wt.% MWCNT loading result up to 10% decrement at both 28 and 90 days curing. Activation energy calculations show fully accordance with these statements, resuming that 0.01 wt.% MWCNT appears to be the most effective loading scheme to produce certain conductivity enhancement in cement mortar.

## 1. Introduction

Concrete is a key material in the construction industry. It is in fact the most used element both in building and infrastructures construction such as bridges, hydraulic works, pavements, etc. Its attributes include easy and affordable manufacturing [1,2], good durability if properly executed [3,4], and remarkable structural capabilities [5,6,7], which make this material the most widely consumed globally. Concrete materials are designed solely to carry compressive loads, although their continuos presence in structures may induce to seek for additional properties which could make concrete a more exploitable material. However, the very nature of cement based materials imposes limitations to develop highly customizable features with added functionalities.

One of the properties that cement microstructure prevents from developing is the electrical conductivity. Wet concrete behaves as a semiconductor, with resistivity in the range of $10^{5}\;\Omega$/mm. However, dry concrete has resistivity in the range of $10^{12}\;\Omega$/mm, which makes the material be considered as an insulator. The variation in the measured electrical resistivity in wet and dry concrete can be interpreted to find that concrete electrical conductivity is an effect of the evaporable water present in the material [8].

The volume of evaporable water found in saturated concrete varies from 60% at the time of mixing to 40% when cement is fully hydrated. This water contains ions whose concentration varies over time, directly affecting concrete conductivity. When the concentration of these ions is very high, ionic association begins, giving rise to C-S-H gel formation and ettringite, which in turns generates an electrical insulation layer in the cement grains. This leads to a decrease in ions mobility, therefore increasing the resistivity. The increase in resistivity with time is also due to less porosity and more tortuosity [9]. After this abrupt rise, resistivity continues to increase at a much slower rate, due to the decrease in hydration reactions. In a porous medium such as concrete, resistivity reflects the ability to transport electric charge throughout the ions dissolved in the aqueous phase of a certain volume, assuming that aggregates are electrically inert since their resistivity turns out to be several orders of magnitude higher than that of the porous solution.

This physico-chemical approach to analyze the capacity to carry electrical charge will not only make it possible to develop a concrete with conductive properties-which will surely lead to very interesting applications-but also, it will provide information on the mechanical performance of the material. Upon mixing cement with water, a suspension is obtained whose resistivity is very low, however, as the cement hydrates and the concrete sets and hardens, the resistivity increases. The evolution of resistivity is therefore parallel to the evolution of strength. In this way, resistivity allows to predicting performance and can act as an indicator of the "age factor", which is essential for certain durability models.

At this point, we incorporate into our study carbon nanotubes (CNTs) within the cementitious material microstructure. CNTs are emerging nanomaterials that have captured the interest of researchers in recent years due to their attractive physical and chemical properties. CNTs mechanical properties, such as strength, stiffness and toughness, have been broadly studied and reported. In particular, Young’s modulus of CNTs is in the range of 1–1.2 TPa, which indicates elastic behavior, with a tensile strength in the order of 36 GPa, denoting yield strains of up to 10% [10,11,12,13,14]. These outstanding mechanical characteristics provide a promising horizon in the achievement of CNT-composites with very high performance strength.

As nanoadditions incorporated into the cement microstructure, CNTs have been widely studied expecting that, due to CNTs superb mechanical performance, an impressive reinforcement in cement-based composites will be achieved. There are indeed a significant number of researchers that have demonstrated great enhancements in flexural and compressive strengths in cement composites by adding low concentration of CNTs. In particular, considering 28 days curing, Li et al. [15] achieved up to 19% and 25% increases in compressive and flexural strength respectively, by adding CNTs 0.5 wt.% to Portland cement paste. Hawreen and Bogas [16] incorporated CNTs additions varying between 0.05% and 0.5% by cement weight to concrete, obtaining improvement in the compressive strength of concrete up to 21%. CNTs were added in ultra high strength concrete by Lu et al. [17], achieving 4.63% increase in compressive strength with 0.05 wt.% CNTs loading. More recently, Hu et al. [18] achieved 2.4% and 9.6% improvements in compressive and flexural strength respectively with the addition of 0.05% CNTs in cement mortar at 28 days curing. However, these researchers reported detrimental effects in compressive and flexural strength with the addition of 0.5% CNTs.

General discussions from these and other reported studies conclude that low loadings of carbon nanotubes are very effective in improving the performance of cement-based materials. However, the incorporation of higher amounts of 0.5% CNTs seems to lead to lower compressive strengths. Furthermore, even the addition of 0.5 wt.% appears to produce contradictory strength results in different cement-based materials, as indicated in the previous paragraph. It seems necessary, therefore, to study in depth the incorporation of CNTs in cementitious materials in order to refine the amounts of CNTs to be added without producing detrimental effects.

However, researchers are clearly in agreement on the existence of a major downside to incorporating CNTs into cementitious composites. CNTs are atomic crystals and therefore do not dissolve in water. Furthermore, the large surface area of nanotubes induces strong attractive forces between the CNTs themselves. These facts result in the formation of clusters and agglomerations of CNTs when mixed with water. These clusters remain even after their insertion into the clinker, due to the van der Waals forces between carbon nanotubes. Furthermore, as CNTs are chemically inert, they do not participate in the hydration process. Consequently, agglomeration of CNTs around the cement grains hinders clinker hydration promoting defects formation in cement composites during the development of their microstructure [19]. All this results in deficiencies in the mechanical properties of the cement-base material, since optimal resistance is developed during the correct hydration of the cement grains. In short, good CNTs dispersion is crucial and helps to obtain maximum mechanical performance by filling the pores and increasing adhesion with cementitious and hydration products [20,21,22,23].

Consequently, some authors have promoted surface modification processes to ensure an adequate dispersion of CNT and a homogeneous inclusion in the cement-water mixture [23,24,25]. Another interesting approach consists of using sonication to achieve a homogeneous dispersion of CNTs in the cementitious matrix. Many authors combine sonication with other shear mixing methods, such as mechanical, magnetic, or/and hand-stirring mixing methods [26,27,28]. On the other hand, cement type influences CNTs dispersion within the matrix, mainly due to the clinker particle size. In fact, most of the tests are carried out with cement of the CEM I 52.5 R type corresponding to Portland cement with a finer particle size and therefore more similar to the size scale of the nanomaterials, as they can be worked together in a more appropriate way. In any case, the hydrophobility of these nanostructures is maintained in the absence of electric field, since the water molecules cannot enter into the graphene layer spontaneously [29]. Consequently, due to their hydrophobility, we can expect that the presence of CNTs in cement microstructure will give rise to movements of evaporable water in the porous structure, and therefore variations in the intrinsic electrical resistivity of the cement composite.

Apart from ionic current due to water presence, electrical conductivity can also be developed in cement microstructure by means of CNTs connectivity, as this nanomaterial exhibits electrical conductivity similar to those of metallic materials [30]. However, it is clear that uniform CNTs distribution is essential to produce a continuous electrically conductive network. In fact, Kim et al. [31] reported that CNTs agglomerations induce damage in the electrically conductive pathways that CNTs should otherwise create. Remarkable improvement in electrical conductivity was achieved by Kim at al. [32], obtaining more than 1000 times reduction in electrical resistivity of well dispersed CNTs-cement paste compared to that of plain cement paste at 28 days curing, whereas poorly-dispersed CNTs-cement paste only achieves electrical resistivity approximately 2 times lower than that of control.

Very large number of CNT modified cement-based materials studies in the current literature aims at assessing the optimal CNT dosage to produce remarkable improvements in strength performance. Despite their importance, these studies use small-scale specimens that are not likely to reflect the actual mechanical behavior of large-scale structures. Taking into account that the electrical resistivity of cement-based materials can be used in quality control or for service life prediction of full size elements, this paper aims at providing more insights at important features that may need to be captured in the ongoing development of standard test methods to be used for CNT modified cement-based materials.

In this work, we present an experimental study on the effect of multi-walled carbon nanotubes (MWCNTs) on the microstructure of cement mortars, in terms of mechanical and electrical properties. For this, standard mortar prismatic samples will be modified with different proportions of MWCNTs, i.e., 0.00 wt.%, 0.01 wt.%, 0.015 wt.%, and 0.02 wt.% by the mass of cement. The nanomaterials will be introduced into the mortar material by priorly prepared water-MWCNT nanofluids following a combined mechanical and sonication dispersion procedure. The consistency, density, setting time and compressive and flexural strength of mixes will be tested and analyzed at 28 and 90 days curing time, in order to account for possible effects due to MWCNT presence in fully hydrated mortar. Finally, this paper will also investigate the electrical resistivity of the different MWCNT-mortar composites and the influence of temperature on the samples resistivity, to confirm the correlation of mechanical and electrical properties of CNT modified cement-based materials.

## 2. Experimental

### 2.1. Materials

#### 2.1.1. Carbon Nanotubes

**Figure 1:**
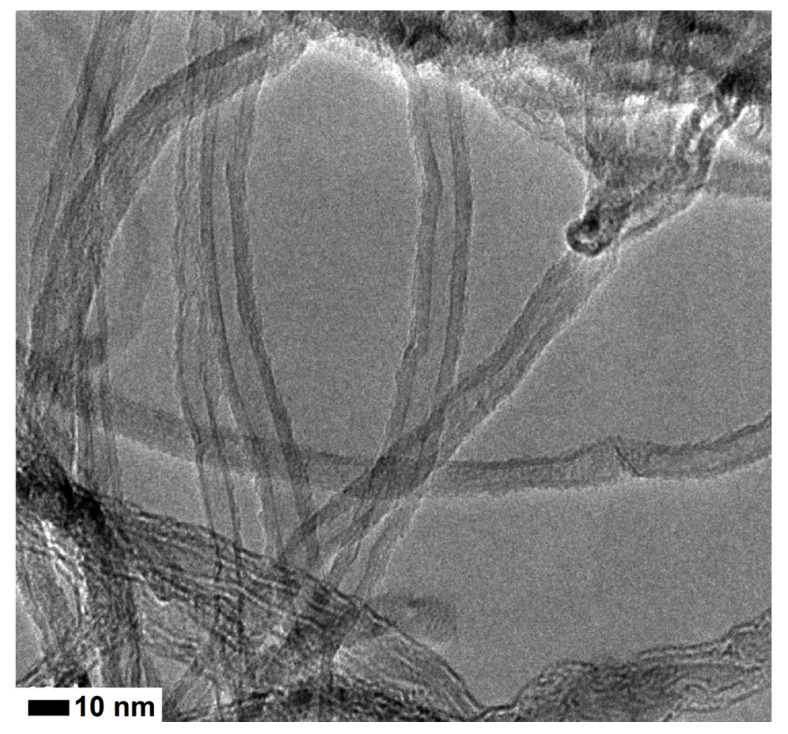
Transmission electron microscope image of MWCNT, adapted with permission from Sikora et al. (2019) [33].

Multi-walled carbon nanotubes, provided by Sigma-Aldrich in Spain, were used. Their properties are given in Table 1. Figure 1 shows its transmission electron microscope (TEM) image [33].

#### 2.1.2. Cement, Sand and Mortar

CEM I 42.5 R type cement was used to manufacture the mortar. Table 2 summarises composition and some physical-chemical properties of the cement used in this work, suppled by Portland Valderrivas, in Spain. Siliceous sand from a nearby quarry, with continuous granulometry and 2 mm maximum size was used. The sand equivalent was 87.35% and the density was 2.63 g/cm${}^{3}$.

Mortar mixes with 0.00 wt.%, 0.01 wt.%, 0.015 wt.% and 0.02 wt.% of MWCNTs were prepared. The mix proportions are shown in Table 3. All samples were mixed in a laboratory drum mixer. Then, the fresh mortar samples were placed in 40 × 40 × 160 mm${}^{3}$ prismatic moulds and homogenized using a vibrating table. Three specimens of each mix were manufactured. The specimens were cured in a curing chamber at 100% humidity and 20 ${}^{\circ }$C temperature. Two curing periods were considered: 28 and 90 days. Additionally, to study the thermal variation of electrical resistivity of MWCNT-mortars, one sample of each MWCNT proportion, for every curing period, was manufactured with a thermocouple inserted at the center of the beam. These specimens were cast and cured in the same conditions as stated before.

Inclusion and dispersion of MWCNTs in the mortar were achieved by prior preparation of MWCNT nanofluids. The corresponding amount of MWCNTs was dispersed in 100 g mixing water by mechanical stirring for 3 min, followed by magnetic stirring for 30 min and sonication for 1 h. Model 505 Sonic Dismembrator (Fisher Scientific, Waltham, MA, USA) was used for sonication. This probe is designed as 20 kHz operating frequency and a maximum power of 500 W. During the sonication, 50% amplitud, and 2 s ON and 2 s OFF pulses were applied. This so-prepared nanofluid was then incorporated to the cement-sand-remain water mix, that kept mixing in the drum mixer for the necessary time (approximately 4 min) until homogeneity.

### 2.2. Methods

#### 2.2.1. Testing Procedures

Technological tests were performed to evaluate the mechanical behavior and durability properties of the cement mortars, namely: consistency, density and setting time of fresh mortars, and flexural strength and compressive strength of hardened mortars at the ages of 7, 28 and 90 days, following UNE-EN 1015-3:2000/A2:2007, UNE-EN 12390-3:2020 and UNE-EN 12390-5:2020 [34,35,36], respectively.

#### 2.2.2. Electrical Resistivity Measurements

The electrical resistivity was measured by means of the four point probe Wenner technique [37] which is an electrical resistivity determination test performed on the surface of the structure or test body. According to Wenner method, by using four equally-distanced electrodes placed on the mortar surface, an alternating current *I* (A) is sent through the two outer electrodes and the voltage *V* (V) is measured through the two inner electrode to finally calculate the apparent resistivity $\rho_{a}$ (kΩ·cm) through Ohm’s law, as expressed in Equation (1)

(1) $$\rho_{a}=\frac{2\pi aV}{I}$$

where *a* (cm) is the distance between electrodes.

This experimental methodology to calculate resistivity assumes that the mortar is semi-infinite and homogeneous. As mortar confined in laboratory specimens cannot be considered semi-infinite, a correction should be made according to the geometry of the specimen [38], by means of a cell constant *K*, so that the real resistivity $\rho_{real}$ is obtained from the following expression given in Equation (2):

(2) $$\rho_{real}=\frac{\rho_{a}}{K}$$

In this work, the electrode spacing used was 2.5 cm, and all measured resistivity values were corrected for the geometry effect with a cell constant K=3.1, according to [39]. An alternating current was applied by a waveform generator (National Instrument, 9263) which imposed a peak amplitude voltage of ±10 V at 500 kHz frequency. Values of the voltage and current intensity were recorded by National Instrument 9227 and 9222 respectively.

**Figure 2:**
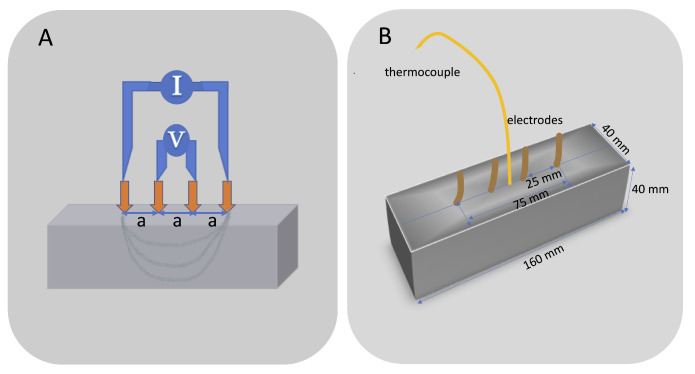
Diagram of Wenner method for resistivity measurements in mortar specimens (**A**), and (**B**) electrode spacing and dimensions.

Figure 2A shows a graphic scheme of the described measurement method. In addition to the thermocouple, four pure copper pins were fixed in the mortar samples surface using a conductive epoxy. Figure 2B shows a schematic of the beam dimensions, spacing and position of the electrodes and temperature probe.

To investigate the electrical transport within the MWCNT-mortar microstructure in terms of activation energy, we introduce the effect of temperature on the resistivity of MWCNT-mortar composites. The resistivity/temperature response is presented in an Arrhenius format thereby allowing evaluation of the activation energy for electrical conduction processes. Specimens were placed in a temperature controlled heater and their resistivity tested over the range 20–60 ${}^{\circ }$C, following the so-called dynamic temperature test (DTT) as described by Liu and Presuel–Moreno [40]. This procedure consists of the monitoring the thermal-electrical behavior of the mortar samples when a thermal gradient is applied. The test was carried out in a controlled environment of constant temperature and relative humidity.

## 3. Results and Discussion

### 3.1. Consistency, Density and Setting Time

Consistency of the fresh MWCNT-mortar samples was determined by flow table. The loading of MWCNTs in mortars resulted in consistency values lower than that of the control mortar. As the MWCNT addition ratio increased, the consistency values decreased (Table 4). However, density values increased as the MWCNT loading increased (Table 4).

Both density and consistency are related to MWCNTs dispersion, since these fresh mortar properties are indicative of homogeneity and porosity. Well dispersed MWCNTs are expected to contribute to better homogeneity and promote filling effects on the porous structure of cement composites. Our results show an increase in density with MWCNTs content, which can be attributed to the reduction of air content in the cement matrix as CNTs are added and occupy empty sites within the pore structure. This itself is an indicative of good MWCNTs dispersion. Coherently, increase in consistency with MWCNTs content should be expected, leading to more fluid mixes as porous structure is refined and mix fine particles get better dispersed. However, taking in account that no superplastifier nor surfactant agent was added along with MWCNTs, the decrease in consistency displayed in our results should be understood as an effect of large water retention in the mortar, mainly due to the higher water-demand of the pastes because of the MWCNTs’ high specific surface.

Regarding the setting time, it was observed that for MWCNT-mortar both the initial and final setting times are shorter than that of the control mortar. Some researchers [41] state the same results. However, initial setting time differences are more significant than final time, although changes in the MWCNT loading did not significantly modify the overall trend. According to [42], this behavior can be explained by a rapid nucleation during the initial steps which later leads to a barrier effect, slowing the formation of additional hydration products in the MWCNT-mortar [14].

### 3.2. Compressive and Flexural Strength

**Figure 3:**
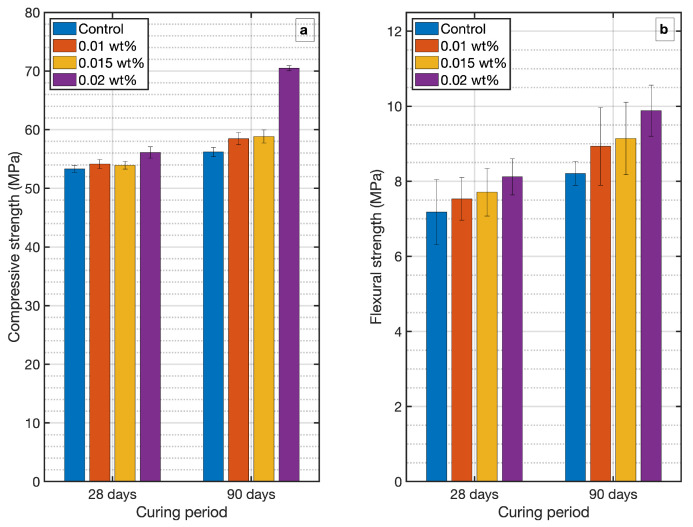
Compressive (**a**) and flexural strength (**b**) of MWCNT-mortar specimens.

The compressive and flexural strength results obtained for each mixture are shown in Figure 3. The maximum failure load and its failure pattern is noted and results are presented in Table 5 below.

**Figure 4:**
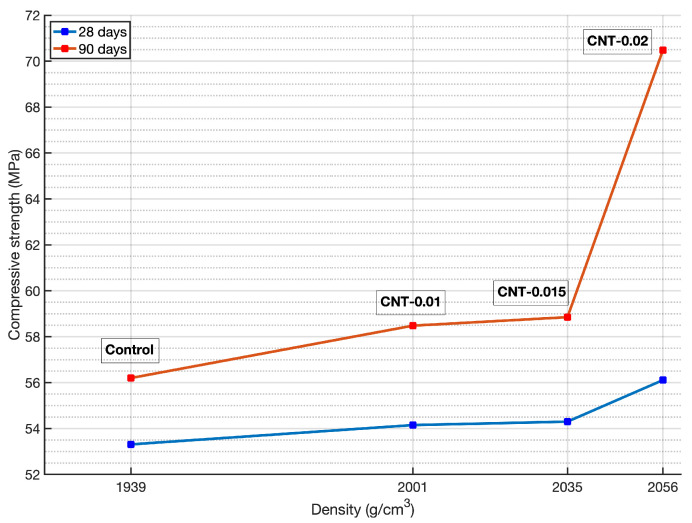
Relationship between compressive strength and density of MWCNT-mortar specimens.

The results confirm an increase in compressive strength in all the specimens with MWCNT content in the cement matrix for the two curing periods, in agreement with related works which also studied low CNT loading [17,43,44]. In particular, all mortars manufactured with 0.01 wt.% MWCNT showed 1.6% and 4.1% higher compressive strength values than those manufactured with ordinary cement (control) for 28 and 90 days curing periods, respectively. Furthermore, the improvements were significant in mortars prepared with 0.015 wt.% MWCNT, which showed 3.0% and 4.7% increases in compressive strength at 28 and 90 days curing period respectively. This increase in strength can be explained by the CNTs-induced formation of highly dense zones in the cement microstructure, as seen in the strength-density relationship shown in Figure 4. The densification of cement, forming virtual aggregate clusters (cement-MWCNTs) with presumably rounded shapes, minimizes crack initiation, propagation and distribution [45]. More remarkable effect was shown in cement mortars with 0.02 wt.% MWCNT loading at the age of 90 days, that exhibited an improvement of 25.4% in compressive strength.

Flexural strength results show the same trends in increasing flexural strength when low MWCNT loading in present in mortar. MWCNT 0.02 wt.% demonstrates to be the optimum dosage in accommodating in the cement matrix, achieving improvements up to 20.3% in flexural strength at 90 days curing period. Additions 0.01 and 0.015 wt.% also showed satisfactory results, with an increase of the ultimate strength of about 8.8% and 11.3%, respectively, at the same curing period of 90 days.

These findings show that the modification of cement microstructure after inclusion of low dosage of MWCNTs results in an increase in the ultimate strength, both compressive and flexural, specially at long curing time. These results also lead to the importance of caring cement paste hydration parameters at long ages, when microstructure development is almost completed, and CNTs can contribute to a filled porous structure with enhanced strength properties. If mortar curing has developed properly under cared curing conditions, hardened material can take advantage of CNTs for an improved loads distribution.

### 3.3. Electrical Resistivity Analysis

**Figure 5:**
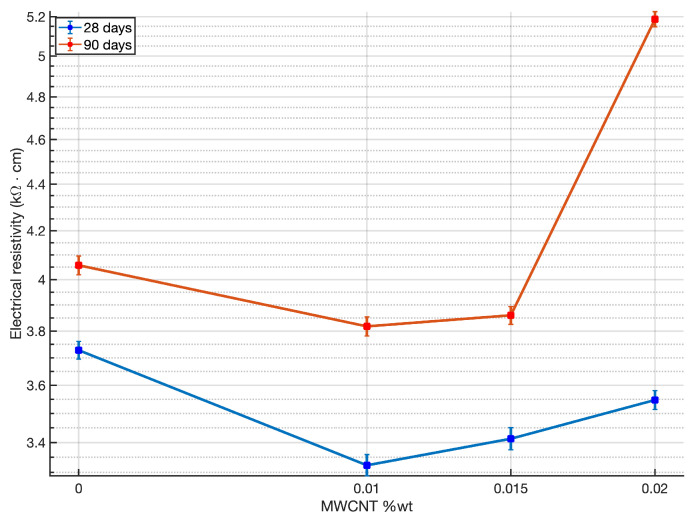
Average electrical surface resistivity of MWCNTs-mortar samples.

Figure 5 shows the average results of the electrical surface resistivity of MWCNT-mortars versus the amount of MWCNTs for each curing time. In agreement with Konsta-Gdoutos and Aza [46], a decrease in resistivity is observed with the presence of MWCNTs in the cement matrix even for very low nanomaterial additions. In general, similar decrement range is observed regarding the curing times, regardless of the MWCNT loading. For example, in the case of mortar specimens with 0.01 wt.%, the registered resistivity values were 3.32 kΩ· cm and 3.82 Ω· cm, at curing times of 28 and 90 days respectively, which corresponds to a decrease of 11% and 6%, respectively. A similar behavior happened in the case of mortar specimens with 0.015 wt.% MWCNT, where electrical resistivity values reached decrements of 8% and 5% at curing times of 28 and 90 days respectively. Thus, in the case of both MWCNT loadings, average values of electrical resistivity are in a similar range, although our findings demonstrate that the curing time does have an influence on the electrical resistivity of the specimens. This statement is in agreement with relevant works where findings on nearly linear increment of resistivity as degree of hydration increases are reported [47,48]. Surprisingly, the sample with the addition of MWCNTs 0.02%, manages to increase the resistivity by 27% at 90 days curing time. These very MWCNT-mortar specimens did show a remarkable increase in compressive strength test at 90 days curing time, which in indeed should explain this different trend at electrical resistivity variation, that is presumed to be a physical property related to mechanical performance of cement-based materials.

Regarding the variation of resistivity with respect to the MWCNTs content in the samples, it is clear from the results that there is a slight detriment in conductivity as the addition of MWCNTs increases. No significant improvement in conduction properties is obtained from an increasing loading of MWCNTs. Once electrical conduction is established in the material, the presence of a greater amount of nanomaterial does not imply a gain in electrical flux. Although in this work we cannot warranty good MWCNTs dispersion within the matrix of our samples, we can derive, however, that good dispersion was achieved from the fresh properties results. Therefore, we believe that conduction paths do not break due to bad MWCNT dispersion, but that resistivity increases due to polarization effects caused by the amount of water and the dissolved ions in the mixes [46], as consistency results proved large water retention in our samples.

**Figure 6:**
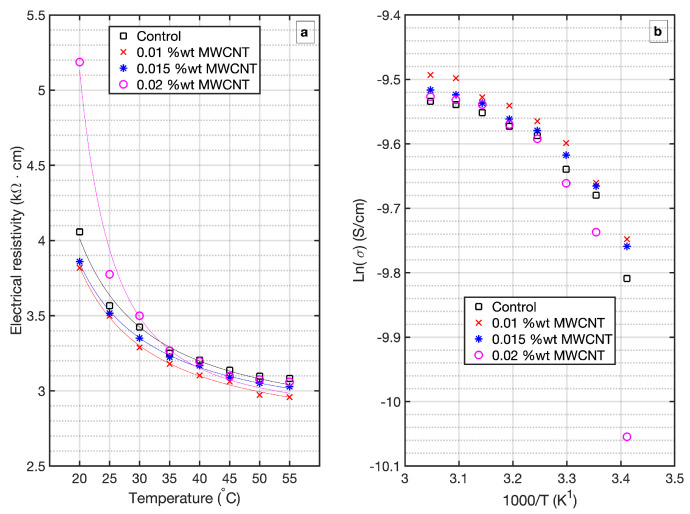
Thermal evolution of electrical resistivity of MWCNT-mortar at 90 days curing time (**a**), and Arrhenius plots for MWCNT-mortar at 90 days curing time presented over the temperature range 20–55 ${}^{\circ }$C (**b**).

Measurements of the dependence of resistivity with temperature are presented in Figure 6a. All samples were tested at the age of 90 days. Temperature significantly influences the measured resistivity, with higher temperatures leading to lower resistivity values, as demonstrated in Figure 6a. From these experimental findings, it is clear that all the profiles of resistivity versus temperature follow a trend of exponential decay, as previously reported [49].

To reach a better insight in this topic, we analyze the activation energy as provided by the Arrhenius equation, given by Equation (3)

(3) $$\rho =A\cdot \exp\frac{E_{a}}{R_{g}T}$$

where ρ is the electrical resistivity measured at temperature T(K), *A* is the pre-exponential factor and represents the nominal resistivity at infinite temperature, $R_{g}$ is Universal Gas constant (8.314 J · mol${}^{-1}$· K${}^{-1}$), and $E_{a}$ is the activation energy for the conduction process (J · mol${}^{-1}$). In Figure 5b below, natural logarithm of the inverse of resistivity (natural logarithm of conductivity σ), is plotted against 1000/T, therefore multiplying the slope of the plot by the Universal Gas constant $R_{g}$, $E_{a}$ is obtained in kJ · mol${}^{-1}$. Results of the calculated activation energy of MWCNT-mortar specimens are presented in Table 6.

The above figures are in agreement with resistivity results as plotted in Figure 6a; high resistivity correlates with high activation energy and vice versa. The activation energy slightly decreases for mortars containing MWCNTs 0.01 and 0.015 wt.%, which implies that in these mortars the energy barrier that must be overcome for an ion to conduct is lesser than the control specimen.

Further understanding of the above facts can be obtained from the analysis of electrical resistivity behavior in porous materials, as mortar, which can be described by using the following Equation (4):

(4) $$\rho_{T}=\rho_{o}\frac{1}{\phi \beta }$$

where $\rho_{T}$ is the total resistivity, $\rho_{o}$ is the resistivity of the pore solution, which in turns is a function of the ions composition and concentration in solution; ϕ is the porosity of the system that is accessible to fluids, and β is the connectivity of the pores in the system [48]. The term 1/ϕβ is ofter called Formation Factor *F*, which comprises the material microstructural characteristics, such as the water-to-cement ratio, volume of paste, and degree of hydration.

Since electrical conduction through saturated cement mortar occurs via ions in the continuous (percolated) aqueous phase between the electrodes [50]; the fact that samples with MWCNT 0.01 wt.% loading show better conductivity than samples with 0.015 wt.% could, in part, be attributed to microstructural changes resulting from further hydration in sample 0.01 wt.% (being hydration reactions thermally activated processes). Another plausible explanation could be the effect of a smaller formation factor in this specimens, which reflects either a higher porosity or a higher connectivity. Higher porosity directly comes out of a more hydrated mortar, as introduced before. Higher connectivity, however, can only be explained by the presence of MWCNTs in the microstructure. From the results, it seems that 0.01 wt.% be more efficient in developing conductive paths within the microstructure. Nevertheless, these findings indicate that the MWCNT loadings studied in this work are insufficient to produce conductive cement mortar.

Similarly, the increase in activation energy, and subsequently increased resistivity, with respect to the control sample for mortars with MWCNT 0.02 wt.% wt can be explained considering that these specimens have better developed their hydration products, as confirmed by the results of mechanical resistance, therefore the formation factor has been enlarged as result of decrease porosity.

## 4. Conclusions

The effect of 0.01, 0.015 and 0.02 wt.% MWCNT loading on mechanical strength and electrical resistivity of cement mortar is studied in the present work. MWCNTs are dispersed in mortar microstructure and tested through mechanical performance analysis at 28 and 90 curing period. The electrical resistivities of MWCNT-mortar are measured at different temperatures up to 60 ${}^{\circ }$C, using the four point probe Wenner setup and dynamic temperature test (DTT). Activation energies corresponding to different MWCNT loadings are calculated using Arrhenius equation.

It is found that an increase in both compressive and flexural strength is achieved in mortars with MWCNT contents. Remarkable improvements of 25.4 and 20.3% at 90 days are found in compressive and flexural strength respectively, for an addition of 0.02 wt.% MWCNTs. Shorter initial and final setting times are obtained for 0.02 wt.% MWCNT loading, which implies a denser microstructure and finer porous structure.

The resistivity measurements in mortars with 0.01 and 0.015 wt.% MWCNT loading result up to 10% decrement to respect to control mortar at both 28 and 90 days curing. Mortar with 0.02 wt.% MWCNTs, however, depicts a sudden 27% increase in resistivity at 90 days curing time, due to well hydrated microstructure as confirmed by the mechanical tests. Activation energy calculations show fully accordance with these statements, resuming that 0.01 wt.% MWCNT seems to be the most effective loading scheme to produce certain conductivity enhancement in cement mortar.

## Figures and Tables

**Table 1:** Properties of multi-walled carbon nanotubes (MWCNTs).

External diameter	6–13 nm
Length	2, 5–20 μm
Size	10 μm
Form	powder
Purity	>98% carbon basis
Special Surface Area	220 m${}^{2}$/g
Density	2.1 g/mL at 25 ${}^{\circ }$C
Average Wall Thickness	7–13 graphene layers

**Table 2:** Elemental composition (wt.%) of source cement and physical-chemical properties (according to the supplier).

Elemental Content (%)	Si	7.78
	Ca	48.2
	K	0.28
	Mg	0.49
	Fe	3.53
	Al	1.32
	P	0.07
	S	1.55
	Ti	0.08
Physical	Blaine number (cm${}^{2}$/g)	3.75
	Setting start time (min)	170
	Initial setting time (min)	170
	Final setting time (min)	220
Loss on Ignition (976 ${}^{\circ }C$)		3.2

**Table 3:** Mortar mix proportions.

Reference	MWCNTs Content (wt.%)	Sand (g)	Cement (g)	Water (g)
Control	0.00	1350	450	225
CNT-0.01	0.01	1350	450	225
CNT-0.015	0.015	1350	450	225
CNT-0.02	0.02	1350	450	225

**Table 4:** Physical properties of the fresh mortars.

Reference	MWCNTs Content (wt.%)	Consistency (mm) UNE-EN 1015-3	Density (g/cm${}^{3}$) UNE-EN 1015-6	Setting Time (min)	
				UNE-EN 196-3	
				Initial	Final
Control	0.00	188.00	1939	140	242
CNT-0.01	0.01	162.50	2001	130	237
CNT-0.015	0.015	158.50	2035	123	228
CNT-0.02	0.02	144.00	2056	119	224

**Table 5:** Compressive and flexural loads and strength.

	MWCNTs Content (wt.%)	Compressive			Flexural		
		Failure	Strength	Increase	Failure	Strength	Increase
		Load (N)	(MPa)	%	Load (N)	(MPa)	%
28 days	0.00	8.74	53.31	–	3.12	7.18	–
	0.01	8.88	54.15	1.6	3.27	7.53	4.9
	0.015	9.0	54.93	3.0	3.35	7.71	7.4
	0.02	9.20	56.11	5.3	3.53	8.12	13.3
90 days	0.00	9.21	56.20	–	3.57	8.21	–
	0.01	9.59	58.48	4.1	3.88	8.93	8.8
	0.015	9.65	58.85	4.7	3.97	9.14	11.3
	0.02	11.55	70.48	25.4	4.30	9.88	20.3

**Table 6:** Summary of activation energy values, calculated with Arrhenius equation of MWCNTs-mortar at 90 days curing time. Conductivity of the MWCNT-mortar at 20 ${}^{\circ }$C, $\sigma_{20}$ is also presented.

MWCNT wt.%	$E_{a}$ (KJ · mol${}^{-1}$)	$\sigma_{20}$× 10${}^{-4}$ (S · cm${}^{-1}$)
Control	8.68	2.46
0.01 wt.%	7.83	2.62
0.015 wt.%	7.38	2.59
0.02 wt.%	17.14	1.93

## Data Availability

The data presented in this study are available on request from the corresponding author.
